# Can Fetal Echocardiographic Measurements of the Left Ventricular Outflow Tract Angle Detect Fetuses with Conotruncal Cardiac Anomalies?

**DOI:** 10.3390/diagnostics11071185

**Published:** 2021-06-29

**Authors:** Alona Raucher Sternfeld, Tal Betzer, Akiva Tamir, Yossi Mizrachi, Sagie Assa, Jacob Bar, Liat Gindes

**Affiliations:** 1 Pediatric Cardiology Unit, Department of Pediatrics, Wolfson Medical Center, Holon 5822012, Israel; alona137@netvision.net.il (A.R.S.); akitamir@gmail.com (A.T.); sagiassa@gmail.com (S.A.); 2 Pediatric Cardiology Clinic, Maccabi Health Services, Rishon-Lezion 7565016, Israel; 3 Department of Obstetrics and Gynecology, Wolfson Medical Center, Holon 5822012, Israel; hagai.tal@gmail.com (T.B.); mizrachi.yossi@gmail.com (Y.M.); jbar@wolfson.health.gov.il (J.B.); 4 Sackler School of Medicine, Tel Aviv University, Tel-Aviv 69978, Israel

**Keywords:** aortic angle, fetus, echocardiography, aortic stenosis, atrio-ventricular canal, transposition of great arteries

## Abstract

Objectives: The angle between the inter-ventricular septum and the ascending aorta can be measured during a sonographic fetal survey while viewing the left ventricular outflow tract (LVOT angle). Our aim was to compare the LVOT angle between fetuses with and without conotruncal cardiac anomaliesrmations. Methods: In this prospective observational study, we compared the LVOT angle between normal fetuses, at different gestational age, and fetuses with cardiac malformations. Results: The study included 302 fetuses screened at gestational age of 12–39 weeks. The LVOT angle ranged from 127 to 163 degrees (mean 148.2), in 293 fetuses with normal hearts, and was not correlated with gestational age. The LVOT angle was significantly wider in fetuses with D-transposition of the great arteries (D-TGA, eight fetuses) and valvar aortic stenosis (AS, three fetuses), than in fetuses with normal hearts (164.8 ± 5.0 vs. 148.2 ± 5.4, respectively, *p* < 0.001). Conversely, the LVOT angle was significantly narrower in fetuses with complete atrioventricular canal defect (AVC, eight fetuses), than in fetuses with normal hearts (124.8 ± 2.4 vs. 148.2 ± 5.4, respectively, *p* < 0.001). On ROC analysis, an angle of 159.6 degrees or higher had a sensitivity of 100% and a specificity of 97.3% for the detection of TGA or AS, whereas an angle of 128.8 degrees or lower had a sensitivity of 100% and a specificity of 99.7% for the detection of AVC defect. Conclusions: The LVOT angle is constant during pregnancy, and differs significantly in fetuses with TGA/AS, and AVC, compared to fetuses with normal hearts (wider and narrower, respectively).

## 1. Introduction

Conotruncal anomalies represent one fifth of all congenital heart defects diagnosed prenatally [1,2]. Prenatal diagnosis leads to a reduction in neonatal morbidity and improved survival, compared to postnatal diagnosis [3,4], and enables expecting parents to perform genetic investigations and to consider termination of pregnancy when indicated. Inclusion of the outflow tract in the basic examination has been reported to increase the detection rates of structural cardiac diseases, from about 50–65% to up to 95%, compared to the four-chamber view alone [5,6]. Although the prenatal diagnosis accuracy of conotruncal anomalies has improved, fetal echocardiography remains a challenging examination^2^, and more screening tools are needed to improve the detection rate of cardiac malformations. 

The objective of this study was to investigate whether the measurement of the angle between the inter-ventricular septum and ascending aorta (LVOT angle) could aid in the identification of fetuses with conotruncal malformations and atrioventricular canal defects. 

## 2. Methods

This was a prospective observational study of singleton pregnancies presenting at the obstetrical ultrasound unit at the E. Wolfson medical center, and at the fetal echocardiography clinic at Maccabi Health Services, between November 2015 and May 2016. The study was approved by the ethical committee of both institutions. All patients signed informed consent prior to the ultrasound examination. Gestational age was determined according to the last menstrual period and/or first trimester ultrasound. Inclusion criteria were singleton pregnancies of which all cardiac structures were demonstrated according to standard guidelines [7,8].

**Figure 1:**
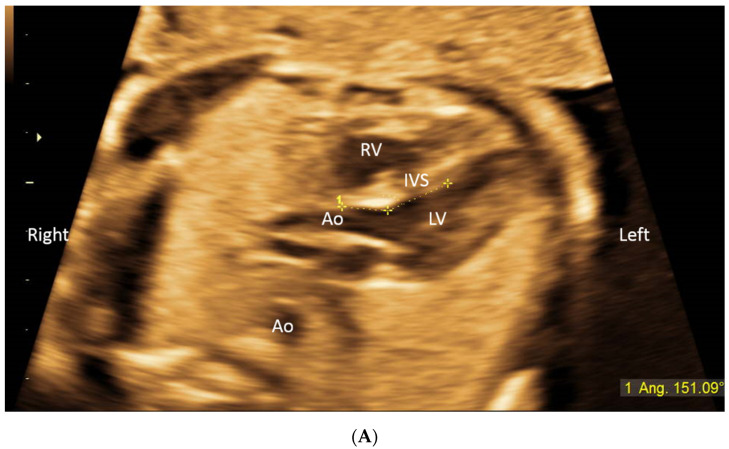

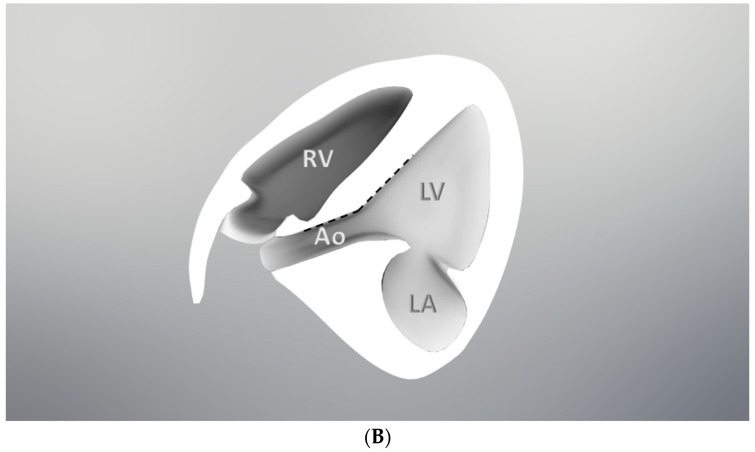
(**A**) LVOT angle measured in a 22 week fetus. (**B**) Drawing of the LVOT measurement. Ao—aorta; IVS—interventricular septum; LV—left ventricle; RV—right ventricle.

The fetal heart was screened in the axial plane, in order to visualize the apex and the aortic outlet. The scanning images and clips were acquired and saved. The LVOT angle from the interventricular septum to the aortic root was measured in the “5-chamber view” [9] at the beginning of a systole, as the aortic valve opens. The LVOT angle for D-TGA cases is the angle with the pulmonary artery and not the aortic root. Measurements were performed on a range of fetal positions, when the apex turned to the transducer, but also with the fetus lying with the back or side to the transducer, while avoiding acoustic shadow (Figure 1, Supplementary Figures S1–S3).

All examinations were performed with Voluson ultrasound machines (E6, E8, E10, GE Healthcare, Kretz Ultrasound, Zipf, Austria) using a 6 MHz transabdominal transducer and a 12 MHz transvaginal transducer, and Vivid 7 or 9 echocardiographic machines (GE Healthcare, Milwaukee, WI), using a C4 MHz transabdominal transducer. 

The examinations were performed by an expert in fetal ultrasonography and fetal echocardiography (author L.G) or a pediatric cardiologist (author A.S.R). Images and clips of the LVOT were saved for later measurements and inter-observer variability, and all were measured again by a resident in ObGyn (author T.B) who was trained accordingly and was blinded to the examination results. 

For statistical analysis we used the mean value of the three measurements of different images taken from the same clip. 

### Statistical Analysis

Data were analyzed using SPSS statistical software v25.0 (IBM ltd., Chicago, IL, USA). Continuous variables were assessed for normality using the Shapiro–Wilk test. Normally distributed variables were compared by the Student’s *t*-test and variables deviating from a normal distribution were compared by the Mann–Whitney U-test. Nominal variables were compared using the chi-square test. All tests were two-sided and considered significant when *p* < 0.05. Inter-observer agreement was assessed using Spearman’s correlation coefficient. Receiver operating characteristic (ROC) analysis was performed in order to calculate the sensitivity and specificity of the LVOT angle for the detection of conotruncal anomalies.

## 3. Results

A total of 302 fetuses with a gestational age of 12–39 weeks were examined during the study period, out of which 19 fetuses were found to have complex cardiac anomalies (eight had complete atrioventricular canal defects, AVC; three had valvar aortic stenosis, AS; eight had complete transposition of the great arteries, D-TGA). Two fetuses with tetralogy of Fallot were excluded because the discontinuity between the septum and the ascending aorta made it difficult to measure the LVOT angle. 

The mean maternal age was 31.8 years (range 20–43 and one patient was 48 years old). We have data on parity for 214 out of 302 patients, about two thirds are multipara (141) and about one third are nullipara (73). About two thirds of the measurements were taken at the second trimester (197 measurements), one third at the third trimester (102 measurements) and only three at the first trimester. 

**Figure 2:**
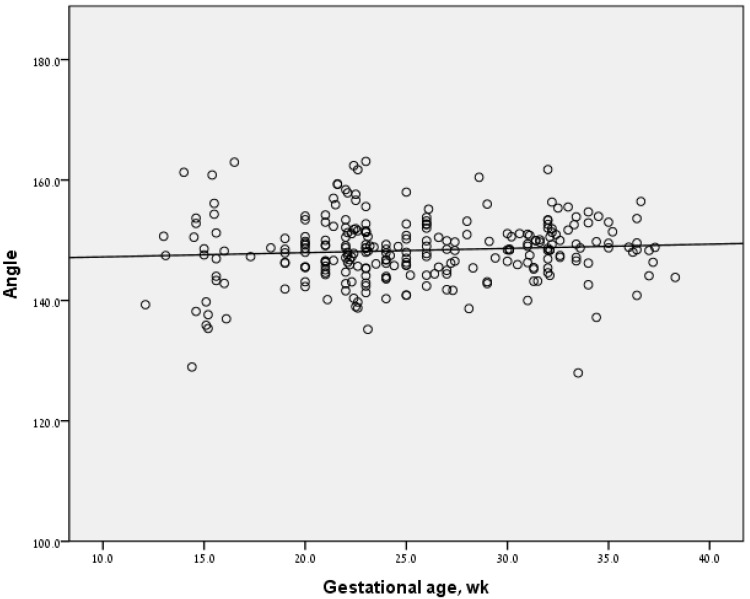
LVOT angle measurements at different gestational ages.

Among fetuses with normal hearts (*n* = 293), angle measurements ranged from 127 to 163 degrees (mean 148.2 ± 5.4). The angle measurement did not correlate with gestational age (Spearman’s correlation coefficient = 0.086, *p* = 0.16), and did not change significantly during pregnancy (Figure 2). Inter-observer agreement was low for normal hearts (Spearman’s correlation coefficient 0.41, *p* < 0.001), but high for fetuses with conotruncal anomalies (Spearman’s Correlation Coefficient 0.73, *p* = 0.037). 

**Figure 3:**
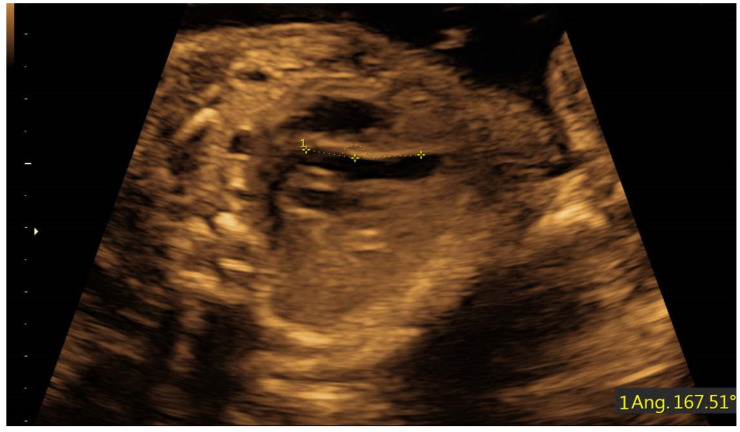
LVOT angle in fetus with D-TGA at 24 gestational weeks.

**Figure 4:**
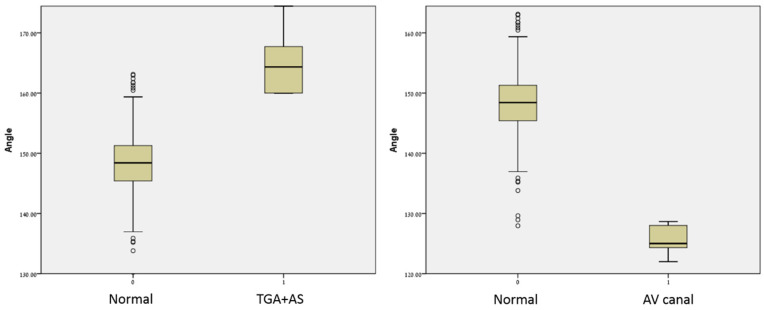
LVOT angles of fetuses with normal hearts, AVC, D-TGA, or AS, measured at different gestational ages.

**Figure 5:**
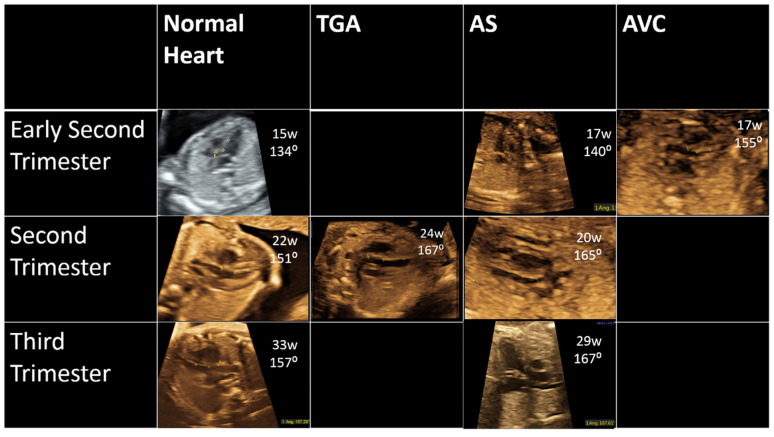
LVOT angle measurements at different gestational ages in the study population.

In fetuses with AVC defects (*n* = 8), angle measurements ranged from 122.0 to 128.6 degrees (mean 124.8 ± 2.4), and was significantly narrower compared to fetuses with normal hearts. In fetuses with D-TGA/AS (*n* = 11) angle measurements ranged from 159.9 to 175.2 degrees (mean 164.8 ± 5.0), and was significantly wider compared to fetuses with normal hearts (Table 1 and Figure 3, Figure 4 and Figure 5). Figure 4 illustrates the distribution of all measurements according to gestational age.

On receiver operating characteristic (ROC) curve analysis, the area under the curve (AUC) was 0.99 (*p* < 0.001) for the detection of TGA/AS. An angle of 159.6 degrees or higher, had a sensitivity of 100% and a specificity of 97.3% for the detection of TGA/AS. The AUC was 0.99 (*p* < 0.001) for the detection of AVC defects. An angle of 128.8 degrees or lower, had a sensitivity of 100% and a specificity of 99.7% for the detection of AVC defect.

As a reference hospital for fetal echocardiography and fetal ultrasound, some of the patients in this study continued their follow-up or gave birth in other medical centers. The outcome of the fetuses with conotruncal anomalies is known for 73% of the study population. One of the TGA fetuses had amniocentesis with normal CMA. The others did not perform any genetic tests. Two of them were born, the diagnosis confirmed, and underwent arterial switch operation immediately after birth. One patient terminated the pregnancy due to other malformations and the autopsy confirmed the prenatal findings. Three patients opted for termination of the pregnancy (all of them before 24 gestational weeks) but refused to autopsy. Three of our AVC patients opted termination of pregnancy without performing genetic tests. Two patients chose to continue the pregnancy with AVC fetus without genetic tests and gave birth to infants with trisomy 21 and AV canal. The three pregnancies with AS had normal CMA. Two of them chose to terminate the pregnancy due to small left heart and one chose to continue the pregnancy and was lost to follow-up. Five patients were lost to follow-up.

## 4. Discussion

The LVOT angle is measurable during fetal cardiac scan at any gestational age. It is wider in fetuses with D-TGA and AS, and narrower in fetuses with AVC defects, compared with fetuses with normal hearts.

**Figure 6:**
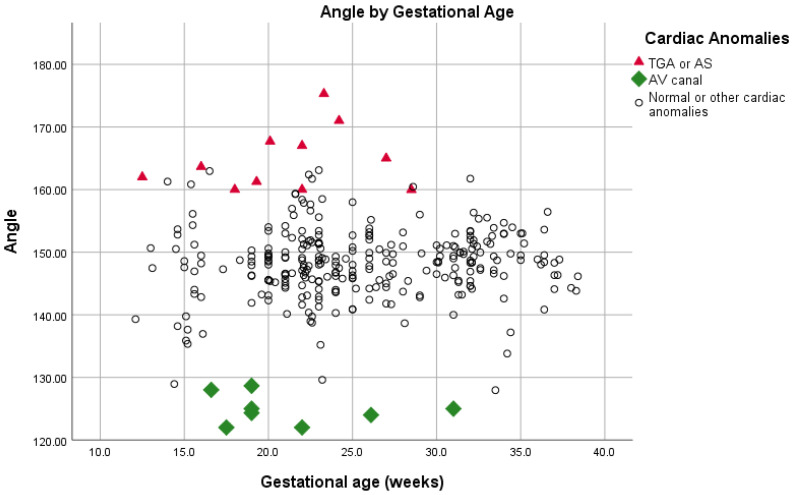
Schematic presentation of the normal LVOT angle with normal variation marked by the green triangle. The red triangle represents the angle seen in cases of AV canal. The orange triangle represents the angle seen in cases of D-TGA or AS.

The normal LVOT angle measured in the study (Figure 6) ranged from 127 to 163 degrees. This wide variation may be explained by the complex spatial shape of the heart, minor inaccuracies of the measurement, and the influence of fetal position on the direction of image acquisition. It should be noted that visually, this variation in the angle is not very large, as demonstrated in Figure 6. Our results are in agreement with previous studies, reporting the normal LVOT angle range in adults to be between 126 and 144 degrees [10], and between 122 and 135 degrees [11], albeit with somewhat different methodology. 

This difference in methodology makes it difficult to compare the LVOT angle measured in adults to that in fetuses. Another study compared the LVOT angle in adult patients with hypertrophic cardiomyopathy and in normal individuals [12]. In this case, the angle was measured between a line drawn along the border of the right and left interventricular septum, parallel to the proximal right ventricular endocardial border, and a line drawn through the long axis of the aortic root. The results from 25 healthy participants gave a mean of 126 ± 6 degrees, while the mean angle in 160 patients with hypertrophic cardiomyopathy was 113 ± 12 degrees. A further study has compared the LVOT angle in 113 adult patients with a sub aortic membrane and 113 matched controls [10]. The angle was measured from the septum to a line draw in the middle of the aorta rather than at the border as we did. In this case, the angles measured in the control patients were between 126 and 144 degrees. Since the described studies each used a different methodology for measurement, we are unable to reach a definite conclusion as to whether the fetus differs from the adult with respect to LVOT and has a different spatial anatomy of the heart. 

Our results indicate that the LVOT angle is more obtuse in fetuses with D-TGA. This reflects the known pathology, since the LVOT angle is measured between the interventricular septum and the main pulmonary artery, which is abnormally posterior to the aorta. In the normal embryologic process, the ventral end of the arterial segment is continuous with the conus, and the dorsal end is continuous with the aortic arches. However, in complete D-TGA, the subaortic portion of the conus persists and the subpulmonary conus is absorbed. The aortic valve then moves anteriorly, and the pulmonary valve moves inferioposteriorly into fibrous continuity with the mitral valve. 

The prenatal detection rate of simple D-TGA is low (3–27%) [13,14,15,16,17]. Whether measurement of the LVOT angle has the potential to improve the diagnosis of D-TGA during screening should be further investigated in a future study. Although a training program was reported to improve the detection rate to 37% [18], without a prenatal diagnosis, the post-natal treatment is delayed, particularly for infants born outside of a tertiary-care center. This is important since in the Netherlands, where the rate of D-TGA detection has improved to 41% since 2012, the mortality rate was found to be lower for patients who were detected prenatally [19]. There were no deaths during the first year for neonates diagnosed prenatally and the patients were found to be in a better condition prior to surgery. 

Our results also revealed that the LVOT angle of patients with AS was more obtuse than found in normal fetuses. This trend was similar to the results reported for an adult population with subaortic membranes compared to normal controls, ~127° vs. 137°, respectively [10]. AS may be caused by abnormal aortic valve or subaortic stenosis. In cases of subaortic stenosis, there is a fibrous crescent-shaped membrane located immediately beneath the aortic valve, or fibromuscular collar [20]. LVOT angle measured in children and adults with subaortic stenosis^11^. The measurement technique was different than ours. They measured the angle from the middle of the thickness of the interventricular septum to the middle of the aorta. They also found more obtuse angle compared to normal in the three group of age they measured (age up to 10 years normal angle of 114–130° and 130–138° for children with subaortic stenosis and intact septum). Since AS may be part of a genetic syndrome (such as Williams–Beuren), an early prenatal diagnosis is important. Again, whether measuring the LVOT angle can improve the rate of detection should be studied further; however, the LVOT angle measurement may provide further support for such diagnosis. 

The reason for the more acute LVOT angle found in cases of AVC is the disproportionate relationship between the inlet and outlet dimensions of the AVC heart compared to a normal heart. The lack of normal atrioventricular septation makes the aorta un-wedged, resulting in an elongated outlet length on the left ventricular surface. The aortic valve is superior to its normal position [21]. 

Angle measurement was not affected by gestational age in our study. This result is expected according to embryological development. At the end of the ninth embryonic week the normal heart is fully formed [22], except for minor morphological features such as the prominent atrial appendages, spiral ventricular arrangement, prominent coronary arteries and thickened arterial walls [23] that appear by the end of 12th embryonic week. 

Our study suggests that LVOT angle measurement is feasible while scanning the fetus. When a broader or sharper angle is found during a sonographic survey, referral for fetal echocardiography is recommended. An LVOT angle larger than 159° raises a suspicion of D-TGA or AS, while an angle smaller than 130° suggests the presence of AVC.

The main limitation of the present study is the low number of fetuses with each of the cardiac anomalies. We did not have enough cases in very early pregnancy, when the nuchal translucency takes place, and this should be in the subject of future studies. Another limitation is that as a referral medical center, especially for fetal echocardiography and obstetrical ultrasound, we do not have the follow-up for most of our patients. 

In conclusion, we suggest that measurement of the LVOT angle should serve as an additional screening tool for fetal cardiac malformations.

## Figures and Tables

**Table 1:** LVOT angles in fetuses with normal hearts as compared with fetuses with AVC and D-TGA or AS.

	Fetuses with Normal Hearts (*n* = 293)	AVC (*n* = 8)	*p*-Value	TGA/AS (*n* = 11)	*p*-Value
Angle	148.2 ± 5.4 (127–163)	124.8 ± 2.4 (122–128.6)	<0.001	164.8 ± 5.0 (159.9–175.2)	<0.001

## Data Availability

The data presented in this study are available on request from the corresponding author.

## Supplementary Materials

The following are available online at https://www.mdpi.com/article/10.3390/diagnostics11071185/s1, Figure S1: Image and clip form normal fetus at 32 weeks, anterior view, Figure S2: Image and clip form normal fetus at 31 weeks, lateral view, Figure S3: Image and clip form normal fetus at 33 weeks, posterior view.
