# Supplementary material for: Can Fetal Echocardiographic Measurements of the Left Ventricular Outflow Tract Angle Detect Fetuses with Conotruncal Cardiac Anomalies?
Source: Diagnostics (Basel). 2021 Jun 29;11(7):1185. doi: 10.3390/diagnostics11071185 (PMC8303209; doi:10.3390/diagnostics11071185)
Supplement: Supplementary file 1 [file diagnostics-11-01185-s001.zip › diagnostics-1264037-supplementary.pdf]

Legends to Supplementary Figures:

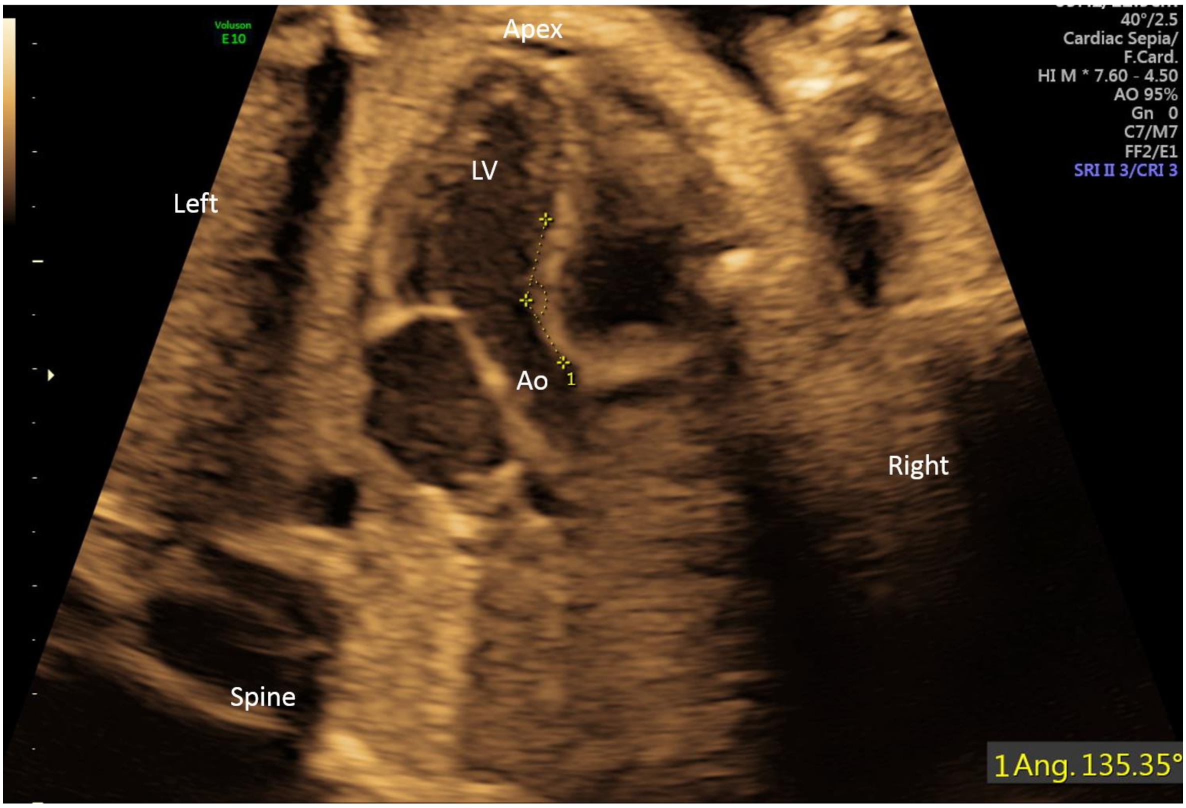

Figure S1 – Image and clip form normal fetus at 32 weeks, anterior view

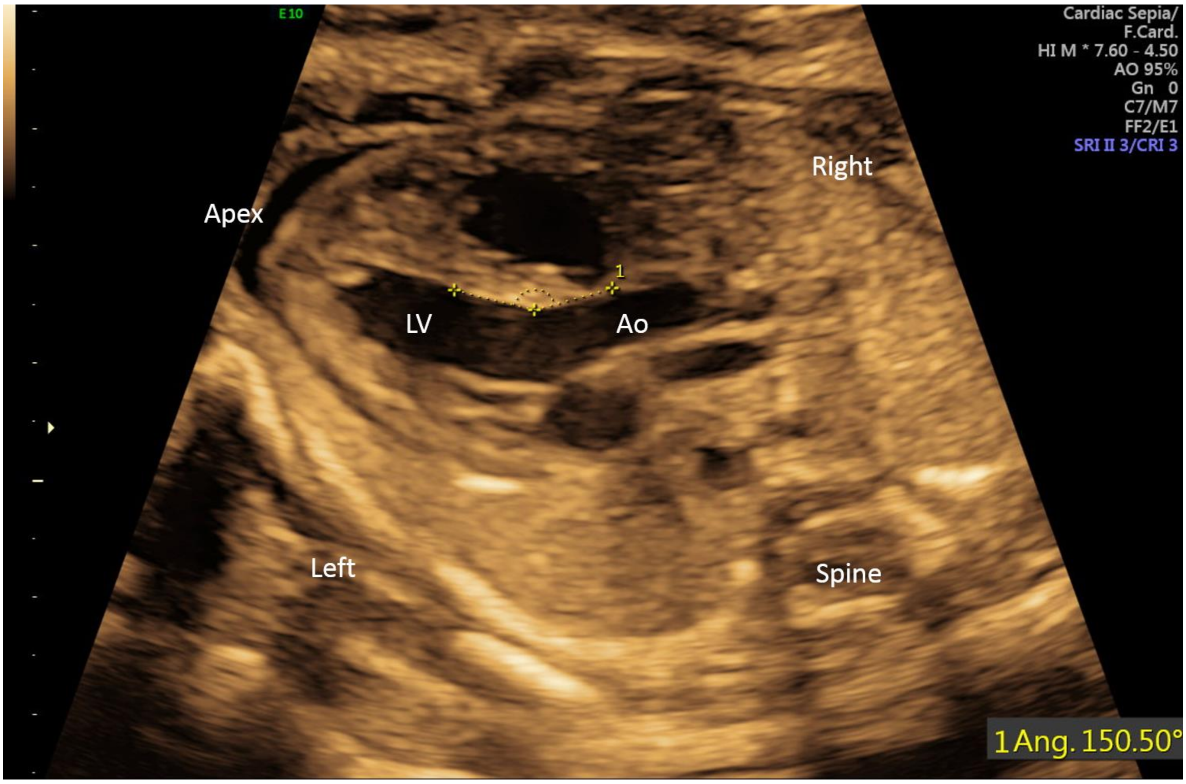

Figure S2 – Image and clip form normal fetus at 31 weeks, lateral view

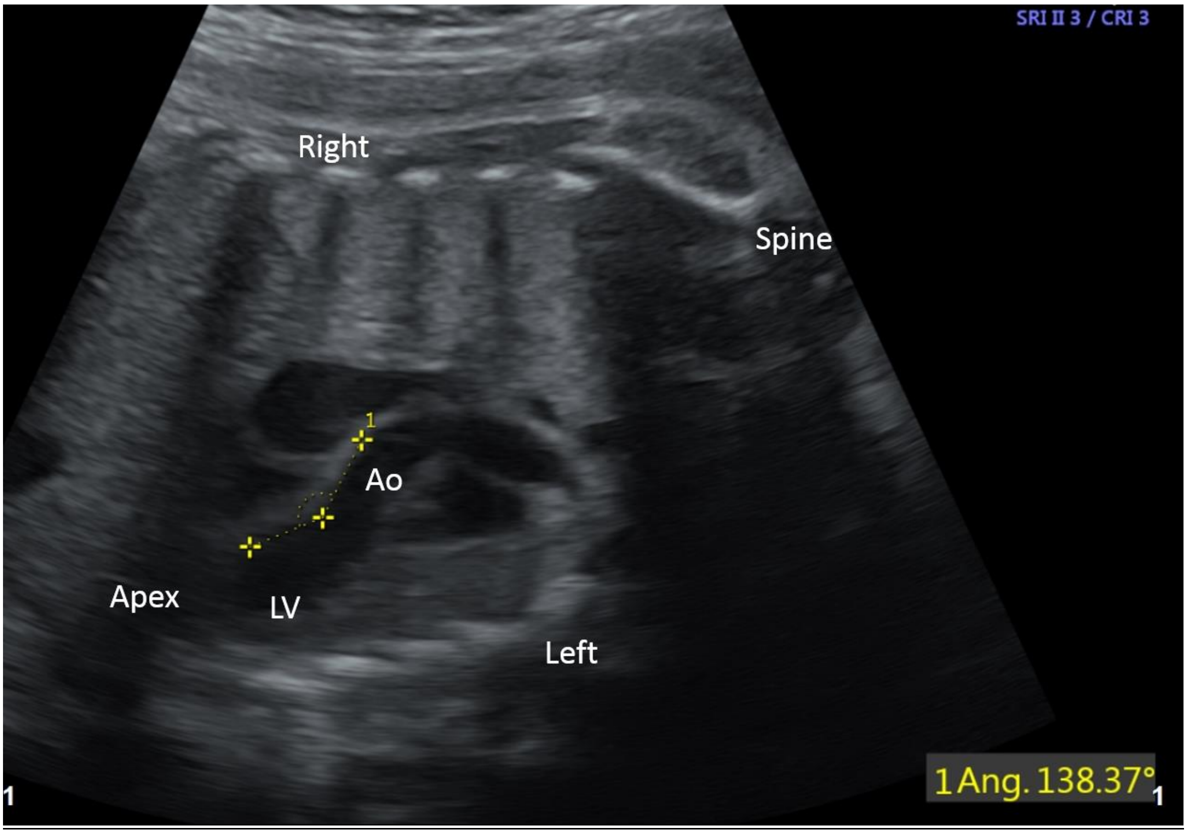

Figure S3 – Image and clip form normal fetus at 33 weeks, posterior view
